# Musculoskeletal Disorders and the Hotel Industry: A Cross-Sectional Survey

**DOI:** 10.7759/cureus.105723

**Published:** 2026-03-23

**Authors:** Ezequiel D Gherscovici, John M Mayer

**Affiliations:** 1 Research and Development, Healthy Buildings LLC, Malibu, USA

**Keywords:** delivery of healthcare, hotel industry, musculoskeletal disorders, patient preferences, survey

## Abstract

Introduction: Musculoskeletal disorders (MSDs) are common, disabling, and challenging to manage. Despite advances in evidence-based practices, clinical outcomes obtained via traditional delivery methods are suboptimal. Previous research suggests that MSD care administered in luxury resort hotels may be useful for some patients, yet large gaps in knowledge exist regarding this delivery approach. The primary objective of this study was to compare the preferences, attitudes, and expectations of healthcare professionals (HCPs) who manage MSDs and members of the general public who do not manage MSDs (Not-HCPs) about receiving MSD services in luxury resort hotels. A secondary objective was to assess perceptions about receiving MSD services in luxury resort hotels versus clinician offices.

Methods: A sample of adults who were identified through convenience sampling from the investigator's professional mailing list and subsequent snowball sampling participated in a cross-sectional survey study. Participants completed an online survey questionnaire one time. The questionnaire, which was developed by the investigators as an exploratory instrument, consisted of 13 items with Likert scale, visual analog scale, dichotomous, multiple choice, and line slider types of questions. Several domains related to MSDs, such as treatment approaches, providers, implementation and adherence factors, and service delivery and locations (clinician's office, luxury resort hotel), were assessed. Analysis was primarily descriptive, and responses from HCPs and Not-HCPs were compared.

Results: 81 respondents completed the survey and had full data sets, of which 43 were originally invited via email (response rate 21.3%) and 38 were from other referrals. The results suggest that, overall, respondents were willing to receive care for MSDs in luxury resort hotels compared with clinician offices. Notably, a high percentage of participants indicated that receiving care for MSDs at a luxury resort hotel would be more comfortable and enjoyable, less intimidating, and more likely to result in adherence to therapeutic exercise and improvement in overall health and wellness. Not-HCPs had more favorable views than HCPs about receiving MSD care in luxury resort hotels, which suggests that perceptions of HCPs and Not-HCPs are misaligned.

Conclusion: The results of this study could be prone to a high risk of bias and low generalizability due to the sampling strategy, small sample size, and exploratory, non-validated nature of the survey instrument. While future research is needed to inform clinical practice, this study provides insights about potential misalignments between perceptions of HCPs and Not-HCPs regarding non-traditional delivery settings for MSDs.

## Introduction

Musculoskeletal disorders (MSDs) are very burdensome, and people of all ages, occupations, and societies around the world are vulnerable to these disorders [1-3]. In comparison to other medical conditions, the Global Burden of Disease study reported that three categories of MSDs (low back pain, neck pain, and other MSDs) resulted in more years lived with disability (YLDs) than diabetes mellitus, chronic obstructive pulmonary disease, ischemic stroke, and ischemic heart disease [3]. Specifically, the rankings for YLDs (with YLDs in thousands, 95% uncertainty interval) for these conditions were low back pain #1 (57648, 40820-75877), neck pain #6 (28936, 19578-40543), other MSDs #7 (28904, 19544-40812), diabetes mellitus #8 (28584, 19534-39575), chronic obstructive pulmonary disease #11 (16288, 14342-17878), ischemic stroke #17 (11802, 8246-15151), and ischemic heart disease #29 (6916, 4812-9385) [3].

Important advancements with evidence-based practices have improved the management of MSDs, and comprehensive clinical practice guidelines are available for many MSD categories, such as low back pain, neck pain, hip pain, and knee pain [4-9]. Typically, evidence-based practices for conservative management of chronic MSDs include physical exercise and education, often within a multimodal treatment plan [4-9]. Specific parameters for delivery of these therapies vary widely and depend on numerous aspects, such as duration of condition, diagnosis, availability of healthcare resources, and other patient-specific factors [4-10].

Despite progress, managing MSDs continues to be challenging and suboptimal across various metrics, including clinical outcomes, prognosis, recovery, implementation factors, healthcare service delivery, costs, adherence, and patient satisfaction [3,11-18]. Low back pain, for example, has been associated with high rates of disability [3] and high levels of recurrence [11]. A cohort study found that within 12 months after initially recovering from an episode of low back pain, 69% of patients had a recurrent episode and 41% received healthcare services for recurrence [11]. Others have described deficiencies in care for low back pain [18], lack of improvements in clinical outcomes despite higher healthcare utilization [13], and wasteful spending [12].

Notably, the implementation of evidence-based guidelines for MSDs within clinical practice has been poor [15], is highly variable [15,17], and has numerous barriers [13], which have contributed to fragmented care and lack of implementation of preventive services [18]. Consequently, the management of MSDs, particularly low back pain, has been linked with suboptimal patient satisfaction [16], and disconnected perceptions about recommendations from clinicians and the goals and expectations of patients [15]. Similarly, adherence to therapies for low back pain is poor [14]. For example, a recent cohort study reported that only 43% of patients adhered to an in-clinic exercise program for low back pain [14].

Considering the substantial areas for improvement and knowledge gaps regarding MSD service delivery, changes in systems and policies should be made [12], and innovations should be explored [19]. Traditionally, healthcare services for MSDs have been delivered in clinicians' offices and related settings [20]. However, recent societal trends have created a substantial consumer demand for efficient and effective delivery methods outside of these traditional settings [19-21]. Among the various possibilities for healthcare service delivery, the hotel industry offers a potential setting for some types of patients with MSDs [19,21].

A recent review assessed treatment of MSDs in hotels and uncovered 10 observational studies and clinical trials that fit eligibility criteria [19]. Five studies examined knee osteoarthritis [22-26], four examined low back pain [27-30], and one examined generalized osteoarthritis [31]. For these studies, spa therapy with or without co-interventions was delivered in luxury resort hotels, and less than half of the studies incorporated interventions that aligned with evidence-based approaches [19]. Although the reviewed studies demonstrated substantial risk of bias and the overall results were inconclusive, findings suggest that delivering healthcare services in luxury resort hotels may be beneficial for some patients with MSDs [19]. Additionally, while the aforementioned studies provide useful information for the field, little is known about the perceptions of receiving MSD services in luxury resort hotels.

The primary objective of this cross-sectional survey study was to compare the preferences, attitudes, and expectations of healthcare professionals (HCPs) who manage MSDs with members of the general public who do not manage MSDs (Not-HCPs) about receiving MSD services in luxury resort hotels. A secondary objective was to assess perceptions about receiving MSD services in luxury resort hotels versus clinicians' offices.

## Materials and methods

Study design

A cross-sectional study was conducted that consisted of a one-time administration of an online survey questionnaire. Cornell University (Ithaca, New York, USA) coordinated regulatory affairs for human subjects, study components related to the graduate student project, and data collection. Healthy Buildings LLC (Malibu, California, USA) coordinated survey instrument development, management of anonymous (de-identified) data, analysis, interpretation, and manuscript preparation. The study is reported according to the Checklist for Reporting of Survey Studies [32], as recommended by the Enhancing the Quality and Transparency of Health Research Network [33]. The checklist for this study is found in the Appendix.

Data collection methods

Questionnaire

The questionnaire (i.e., survey instrument) is shown in the Appendix. A 13-item questionnaire was administered to assess the preferences, attitudes, and expectations of HCPs and Not-HCPs about receiving MSD services in luxury resort hotels compared with clinicians' offices. Various question types, scales, and response formats were incorporated into the questionnaire, for which templates are provided by the Qualtrics platform (Qualtrics, LLC, Provo, UT) [34]. Depending on the item's intent and concepts assessed, formats included dropdown menu (survey item number 1), dichotomous (yes, no) (survey item numbers 2, 3, 6, 7), ordinal Likert scale (4- to 7-point) (survey item numbers 4, 5), visual analog scale (0-10) (survey item numbers 8, 12), multiple choice (6-point) (survey item number 9), and line slider (0%-200%, 0-21 days, $0-$1000) (survey item numbers 10, 11, 13) [35-37]. When considering all sub-components of the 13 items, the questionnaire cumulatively consisted of 51 questions. All participants were asked to complete 11 items, while HCPs were asked to complete two additional items. Given the exploratory nature of the study, the reliability and validity of the questionnaire were not formally assessed.

Questionnaire Development

The questionnaire, including its concepts, structure, item characteristics, content for stem and response options, and instructions, was originally developed by the investigators. For the present study, the questionnaire was designed to serve as an exploratory, non-validated instrument to assess a wide array of domains related to MSDs in HCPs and Not-HCPs, such as treatment approaches, providers, implementation and adherence factors, and service delivery and locations (clinician's office, luxury resort hotel). Clinician's office, luxury resort hotel, and MSDs are operationally defined in the questionnaire as depicted in the Appendix. The operational definitions of HCP and Not-HCP were determined by the survey item 2, "Are you a HCP who manages MSDs?" (Appendix). "Yes" responses to this item were categorized as HCP, while "No" responses were categorized as Not-HCP. The investigators assumed that the participants had the ability, training, and experience to self-identify as a HCP or Not-HCP, based on this survey item. Information about sub-groups of HCPs was not sought for pragmatic reasons (e.g., keeping the number of survey items manageable for the participant), and assessment of sub-groups of HCPs was not the aim of the study.

Following initial development, the alpha version of the instrument was reviewed by nine professionals with extensive experience in pertinent fields, including specialists involved with management of MSDs (clinical, research, program implementation, medical billing/coding), along with real estate, academic, and hospitality stakeholders. A formal consensus process was not used during review of the alpha version of the instrument. Based on feedback from the professionals, the investigators developed a beta version of the instrument that was uploaded to the Cornell University version of the Qualtrics software platform [38], and was used for pilot testing. The beta version was administered over 100 times by the investigators, professionals mentioned above, and individuals who were representative of the target population. Based on observations during pilot testing, the instrument was modified by the investigators to establish a final version, which was tested for quality assurance before its release as the live version for use in the study. The investigators reviewed the online instructional training resources for Qualtrics [38], and no additional training was conducted.

Sample characteristics

A sample of adults, who were identified through convenience sampling from the investigator's professional mailing list and subsequent snowball sampling, were invited to participate in this study. Inclusion criteria for participation were being ≥18 years of age and having access to complete an internet-based survey. The data selection process consisted of reviewing surveys for validity and completeness. Respondents with valid and complete surveys (i.e., full data sets) were included in the analysis, while respondents with invalid or incomplete surveys were excluded.

Participants were recruited in two ways. First, candidates for participation were identified from a convenience sample derived from the principal investigator's mailing list of professional colleagues and other acquaintances. An anonymous list of email addresses was uploaded to Qualtrics, which allowed the generation of a generic, shareable URL link rather than tracking individual invitations and user data. An email message was sent from Qualtrics to individuals on this list, which invited them to participate, and included a URL link to access the study's purpose, consent, instructions, and questionnaire. In addition to this distribution mechanism, the email asked the original recipient to forward the email message to other adults, which served as an additional recruitment strategy through snowball sampling. Thus, the sample consisted of participants from two distribution channels: originally invited through convenience sampling and other respondents through snowball sampling. No additional advertising for recruitment was conducted.

Given the exploratory nature of the study, no formal sample size calculation or power analysis was conducted. The investigators acknowledge that uncontrolled convenience sampling (versus random sampling), along with a relatively small sample size, may have resulted in a non-representative sample that was prone to high risk of bias and low generalizability (i.e., poor external validity) [39]. Nevertheless, the study's sampling technique was sufficient for its aim and to inform future research.

Survey administration

The survey was accessible via a URL link that was embedded in the recruitment email, which provided access to the consent, instructions, and survey. The survey was electronically administered by Qualtrics [38] in a private location determined by the participants. If candidates did not respond to the first email, the recruitment email was sent two additional times (three total). The first email request was sent on April 14, 2025, and the last request was sent on August 14, 2025. The first response (i.e., completed survey) was submitted on April 14, 2025, and the last response was submitted on August 27, 2025. The survey was designed to be completed in approximately 10 to 15 minutes.

Qualtrics includes various safeguards in the survey administration processes to protect privacy, data security, and quality of responses [40-43]. For example, to restrict the possibility of unanswered questions, the respondent was unable to move forward to subsequent questions without completing prior questions. Multiple attempts to complete the survey by the same person were restricted and tracked to determine unique visitors by documenting the IP address from which the respondent completed the survey, not allowing multiple attempts for the same IP address, and establishing a unique response ID and external reference ID for each response. A "reCaptcha score" is calculated to "detect the likelihood that a response came from a bot rather than a human subject" [40]. Response quality is validated by examining completion time, completion rate, outliers, straight lining, and vague text for free-form fields. To protect the privacy of participants, IP address, response ID, and external reference ID are anonymized by Qualtrics and are unavailable to investigators. For data security and encryption, Hypertext Transfer Protocol Secure (HTTPS) with Transport Layer Security (TLS) encryption is used for data transmission, login sites are password-protected, and servers and data centers are audited according to independent industry standards.

Ethical considerations

The protocol was granted exemption from institutional review board (IRB) review according to the policy of Cornell University (Ithaca, New York) IRB for human participant research and under the Department of Health and Human Services Code of Federal Regulations 45CFR46.104(d) (Cornell IRB Protocol Number: IRB0149624, Notice of Exemption: March 14, 2025). Each participant provided informed consent, whereby completion of the survey was considered consent. Participation was voluntary, and participants could withdraw from the study (i.e., discontinue completion of the survey) at any time, without penalty. No personally identifiable information was collected, and individual responses were kept confidential from investigators and Cornell University staff with access to Qualtrics.

Analysis

Response quality of the data was examined for completion time, completion rate, outliers, straight lining, and vague text for free-form fields [40]. Following examination of response quality, analysis of the final data was generally descriptive, which was consistent with the study's exploratory aim. Response rates were calculated for the distribution channel of respondents who were originally invited via email generated from Qualtrics. Data from respondents with complete surveys and full data sets were used for analysis. Descriptive statistics were calculated for categorical variables (e.g., frequency, percent) and continuous variables (e.g., mean, standard deviation). The respondents' self-identified HCP status (HCP or Not-HCP) was determined through survey item 2, which was a dichotomous (yes, no) variable. Comparisons between respondents who were HCPs who manage MSDs and members of the general public who do not manage MSDs (Not-HCPs) were made using chi-square analysis for categorical variables and independent T-tests for continuous variables. Specifically, chi-square was used for variables with dichotomous (yes, no) (survey item numbers 3, 6, 7), ordinal Likert scale (4- to 7-point) (survey item numbers 4, 5), visual analog scale (0-10) (survey item numbers 8, 12), and multiple choice (6-point) (survey item number 9) response formats. Independent T-tests were used for variables with line slider response formats (survey item numbers 10, 11, 13). Data analysis was conducted using Stata 7.0, 2002 (Stata Corp., College Station, TX). Statistical significance was set at alpha = 0.05 for all analyses.

## Results

Respondent characteristics

**Figure 1:**
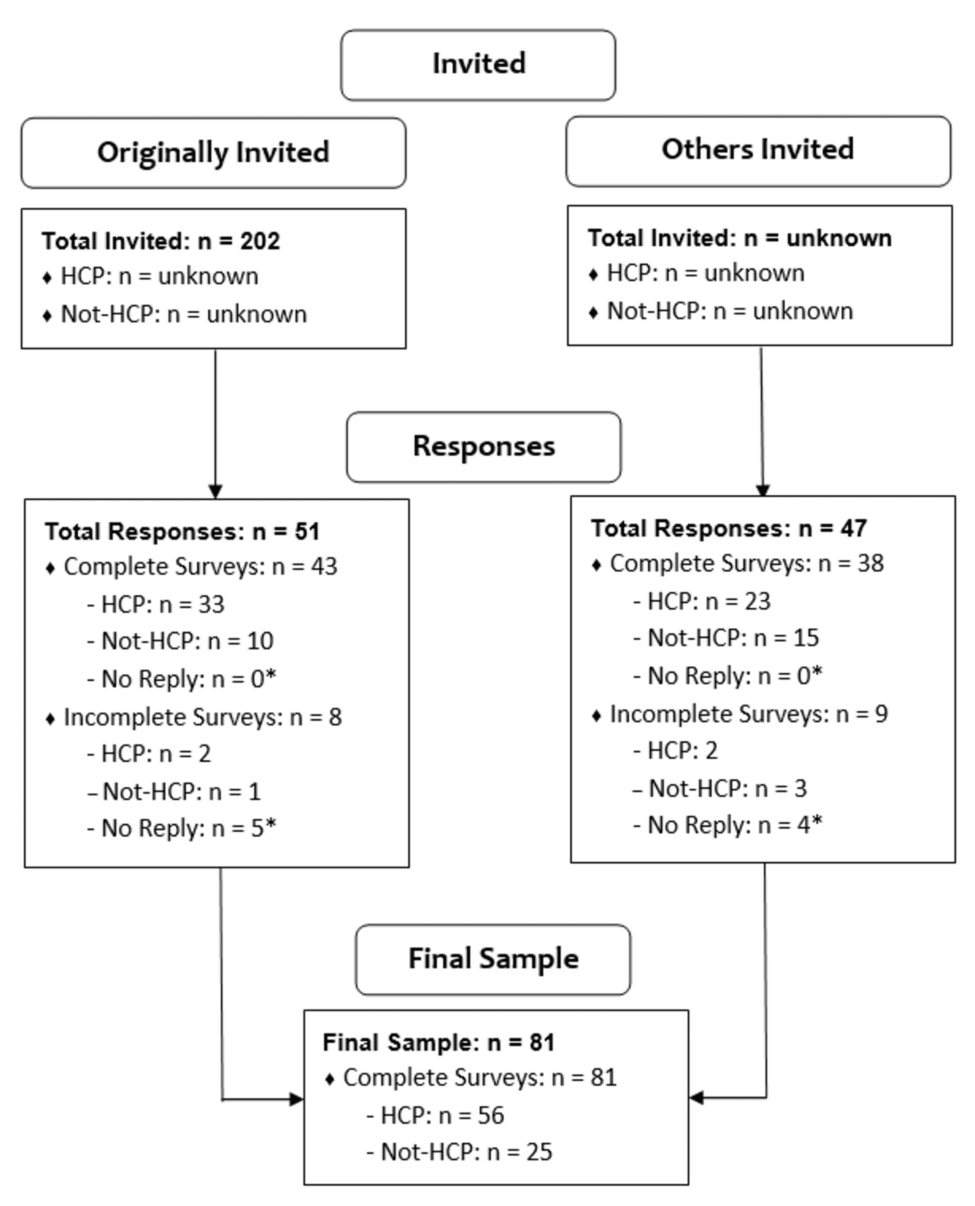
Flow diagram of respondent enrollment. Values listed in number of responses (n). Originally invited: Individuals who were invited to participate via an original email invitation from investigators (convenience sampling). Others invited: Individuals who responded to the survey via a forwarded invitation from others (snowball sampling). HCP: Healthcare professional who manages musculoskeletal disorders (MSDs). Not-HCP: Member of the general public who does not manage MSDs. * No reply: respondents with incomplete surveys who did not reply to HCP status (yes, no) item.

Figure 1 depicts participant enrollment numbers stratified by distribution channel (originally invited, others invited), HCP status (yes - HCP, no - Not-HCP), and survey completion status (yes, no). For those who were originally invited via email (n = 202), 51 responded (response rate: 25.2%, 51/202), of which 43 had complete surveys (response rate: 21.3%, 43/202). For others who were invited, 47 responded, and 38 had complete surveys. Hence, 81 respondents had complete surveys with full data sets, and 17 respondents had incomplete surveys. The completion rate was 82.7% (81/98), which was considered reasonable by the investigators.

For respondents with complete surveys (n = 81), the time to complete the survey was a median of 6.4 minutes and varied widely, ranging from 3 minutes to 4,377 minutes. Outliers were identified in which the survey was completed abnormally fast ("speeders") [40] and abnormally slow. No significant difference in completion time was observed between HCPs and Not-HCPs (p = 0.481). For respondents with incomplete surveys (n = 17), the percentage of completed items was as follows: 3% of completed items (n = 3), 6% of completed items (n = 4), 10% of completed items (n = 2), 16% of completed items (n = 4), 45% of completed items (n = 2), and 81% of completed items (n = 2).

There was no evidence of straightlining of responses or ambiguous text for free-form fields. Except for completion time, no other types of outliers were identified that could impact response quality and validity. Since the sample size for completed surveys (n = 81) was below 100, outliers based on completion time were included in the analysis [40]. Hence, the final sample size used for analysis consisted of 81 respondents.

Descriptive results

For respondents with complete surveys, 69.1% (56/81) were HCPs and 30.9% (25/81) were Not-HCPs. 84.0% (68/81) had a lifetime history of receiving services from an HCP for MSDs, and 16.0% (13/81) had not received these services. No significant difference (p = 0.994) in the lifetime history of receiving MSD services was observed between HCPs and Not-HCPs.

The 81 respondents represented 20 countries, including seven countries from America; eight from Europe; three from Asia, and two from Oceania, as follows: USA (n = 38), Argentina (n = 10), New Zealand (n = 5), Australia (n = 3), Canada (n = 3), Chile (n = 2), Israel (n = 2), Italy (n = 2), Mexico (n = 2), Spain (n = 2), Uruguay (n = 2), Brazil (n = 1), Czech Republic (n = 1), Denmark (n = 1), France (n = 1), Germany (n = 1), India (n = 1), Japan (n = 1), Netherlands (n = 1), and Switzerland (n = 1).

Main results

Table 1 depicts the respondents' perceptions about receiving MSD services at a luxury resort hotel versus a clinician's office, under the assumption that each facility type is equal in terms of having appropriately trained healthcare staff, rehabilitation equipment, other resources needed to safely deliver healthcare services, and costs. For all eight components of this item, significant associations (p < 0.05) were found between perceptions about receiving MSD services at a luxury resort hotel and HCP status. Overall, Not-HCPs had more favorable perceptions about receiving MSD services at a luxury resort hotel than HCPs. For example, a higher percentage of Not-HCPs reported "agree" or "strongly agree" compared with HCPs. The Not-HCPs had a higher percentage of "agree" or "strongly agree" than "disagree" or "strongly disagree" for 7/8 components of this item. In contrast, the HCPs had a higher percentage of "disagree" or "strongly disagree" than "agree" or "strongly agree" for 6/8 components of this item.

**Table 1:** Respondents' perceptions about receiving services for MSDs. Specific survey question: "Select the response that most accurately represents your expectations about receiving services for a MSD." Tabulation (n, %) of original survey data from respondents. Completion of the survey by the respondent was considered informed consent. HCP: healthcare professional who manages musculoskeletal disorders (MSDs). Not-HCP: Member of the general public who does not manage MSDs. p: Probability value from chi-square analysis for the association between perceptions and HCP status, which assessed the difference between HCP and Not-HCP. *p < 0.05.

Variable	HCP Status				Total		p
	HCP		Not-HCP				
	n	%	n	%	n	%	
I would rather go to a luxury resort hotel for treatment of an MSD than a clinician's office.							0.018*
Strongly disagree	11	19.64	3	12	14	17.28	
Disagree	11	19.64	0	0	11	13.58	
Neither agree nor disagree	24	42.86	10	40	34	41.98	
Agree	8	14.29	8	32	16	19.75	
Strongly agree	2	3.57	4	16	6	7.41	
I believe that a luxury resort hotel is more convenient for me than a clinician's office.							0.020*
Strongly disagree	12	21.43	2	8	14	17.28	
Disagree	22	39.39	5	20	27	33.33	
Neither agree nor disagree	18	32.14	11	44	29	35.8	
Agree	2	3.57	1	4	3	3.7	
Strongly agree	2	3.57	6	24	8	9.88	
I believe that a luxury resort hotel will be more comfortable for me than a clinician's office.							0.003*
Strongly disagree	4	7.14	0	0	4	4.94	
Disagree	7	12.5	0	0	7	8.64	
Neither agree nor disagree	16	28.57	1	4	17	20.99	
Agree	20	35.71	14	56	34	41.98	
Strongly agree	9	16.07	10	40	19	23.46	
I believe that a luxury resort hotel will be less intimidating for me than a clinician's office.							0.003*
Strongly disagree	11	19.64	1	4	12	14.81	
Disagree	12	21.43	3	12	15	18.52	
Neither agree nor disagree	20	35.71	4	16	24	29.63	
Agree	8	14.29	8	32	16	19.75	
Strongly agree	5	8.93	9	36	14	17.28	
I believe that a luxury resort hotel is more likely to help my disability and pain than a clinician's office.							0.039*
Strongly disagree	11	19.64	1	4	12	14.81	
Disagree	16	28.57	3	12	19	23.46	
Neither agree nor disagree	24	42.86	15	60	39	48.15	
Agree	4	7.14	3	12	7	8.64	
Strongly agree	1	1.79	3	12	4	4.94	
I believe that a luxury resort hotel is more likely to improve my overall health and wellness than a clinician's office.							0.003*
Strongly disagree	7	12.5	1	4	8	9.88	
Disagree	14	25	1	4	15	18.52	
Neither agree nor disagree	25	44.64	8	32	33	40.74	
Agree	7	12.5	12	48	19	23.46	
Strongly agree	3	5.36	3	12	6	7.41	
I believe that I am more likely to adhere to exercise recommendations at a luxury resort hotel than a clinician's office.							< 0.001*
Strongly disagree	9	16.07	1	4	10	12.35	
Disagree	14	25	3	12	17	20.99	
Neither agree nor disagree	23	41.07	4	16	27	33.33	
Agree	7	12.5	14	56	21	25.93	
Strongly agree	3	5.36	3	12	6	7.41	
I would more likely enjoy the experience of receiving MSD services at a luxury resort hotel than a clinician's office.							0.017*
Strongly disagree	4	7.14	1	4	5	6.17	
Disagree	4	7.14	1	4	5	6.17	
Neither agree nor disagree	15	26.79	0	0	15	18.52	
Agree	23	41.07	12	48	35	43.21	
Strongly agree	10	17.86	11	44	21	25.93	

Table 2 depicts the respondents' perceptions regarding factors influencing decisions about receiving MSD services at a luxury resort hotel. No significant associations (p > 0.05) were found between factors influencing decisions about receiving MSD services at a luxury resort hotel and HCP status for any of the factors of this item. Overall, the respondents reported that most factors were important to influence their decisions, with 7/8 factors displaying a higher percentage of "very important" or "extremely important" responses than "slightly important" or "not important at all."

**Table 2:** Respondents' perceptions regarding factors influencing decisions about receiving services for MSDs at a luxury resort hotel. Specific survey question: "How important are each of these items for you when making decisions about receiving services for MSDs at a luxury resort hotel?" Tabulation (n, %) of original survey data from respondents. Completion of the survey by the respondent was considered informed consent. HCP: Healthcare Professional who manages musculoskeletal disorders (MSDs). Not-HCP: Member of the general public who does not manage MSDs. MSD: musculoskeletal disorder. p: Probability value from chi-square analysis for the association between perceptions and HCP status, which assessed the difference between HCP and Not-HCP. *p < 0.05.

Variable	HCP Status				Total		p
	HCP		Not-HCP				
	n	%	n	%	n	%	
Proximity (i.e., travel time) of the hotel to my residence.							0.934
Not important at all	1	1.79	1	4	2	2.47	
Slightly important	3	5.36	1	4	4	4.94	
Of average importance	17	30.36	9	36	26	32.1	
Very important	22	39.29	8	32	30	37.04	
Extremely important	13	23.21	6	24	19	23.46	
Receiving some type of incentives for these services.							0.104
Not important at all	16	28.57	6	24	22	27.16	
Slightly important	4	7.14	0	0	4	4.94	
Of average importance	22	39.29	17	68	39	48.15	
Very important	11	19.64	2	8	13	16.05	
Extremely important	3	5.36	0	0	3	3.7	
Understanding if my insurance covers these services or if they are paid for out of my pocket.							0.081
Not important at all	1	1.79	2	8	3	3.7	
Slightly important	3	5.36	1	4	4	4.94	
Of average importance	9	16.07	1	4	10	12.35	
Very important	26	46.43	7	28	33	40.74	
Extremely important	17	30.36	14	56	31	38.27	
Being able to afford these services.							0.376
Not important at all	0	0	0	0	0	0	
Slightly important	0	0	1	4	1	1.23	
Of average importance	4	7.14	1	4	5	6.17	
Very important	29	51.79	15	60	44	54.32	
Extremely important	23	41.07	8	32	31	38.27	
Understanding the recovery time from MSD to estimate my length of stay at the luxury resort hotel.							0.276
Not important at all	3	5.36	1	4	4	4.94	
Slightly important	0	0	0	0	0	0	
Of average importance	8	14.29	1	4	9	11.11	
Very important	28	50	18	72	46	56.79	
Extremely important	17	30.36	5	20	22	27.16	
Determining how to find a luxury resort hotel that provides MSD services.							0.063
Not important at all	4	7.14	1	4	5	6.17	
Slightly important	4	7.14	1	4	5	6.17	
Of average importance	16	28.57	1	4	17	20.99	
Very important	24	42.86	14	56	38	46.91	
Extremely important	8	14.29	8	32	16	19.75	
Assessing the caliber of the healthcare professionals providing MSD services.							0.405
Not important at all	0	0	0	0	0	0	
Slightly important	0	0	0	0	0	0	
Of average importance	2	3.57	0	0	2	2.47	
Very important	22	39.29	13	52	35	43.21	
Extremely important	32	57.14	12	48	44	54.32	
Understanding if my MSD can be treated at a luxury resort hotel.							0.497
Not important at all	4	7.14	0	0	4	4.94	
Slightly important	0	0	0	0	0	0	
Of average importance	6	10.71	2	8	8	9.88	
Very important	25	44.64	11	44	36	44.44	
Extremely important	21	37.5	12	48	33	40.74	

Table 3 depicts the respondents' expectations about MSD services provided at a luxury resort hotel but not at a clinician's office. For 12/16 components of this item, no significant associations (p > 0.05) were found between expectations about MSD services provided at a luxury resort hotel and HCP status. For 4/16 components (Synchronized scent, sound, and visuals; Healthy building certification; Sustainable practices; Crystal healing areas), significant associations were found between expectations about MSD services provided at a luxury resort hotel and HCP status. For these four items, a higher percentage of Not-HCPs reported "Yes" compared with HCPs. Overall, more than 50% of respondents reported "Yes" for 5/16 items, indicating that they would expect to see this feature at a luxury resort hotel but not at a clinician's office, with relaxing ambiance (80.25%) and exceptional customer service (70.37%) ranking the highest.

**Table 3:** Respondents' expectations about MSD services provided at a luxury resort hotel versus a clinician's office. Specific survey question: "What features and services do you expect to see at a luxury resort hotel that you will not find at a clinician's office when receiving MSD services? Select all that apply." Tabulation (n, %) of original survey data from respondents. Completion of the survey by the respondent was considered informed consent. HCP: Healthcare professional who manages musculoskeletal disorders (MSDs). Not-HCP: Member of the general public who does not manage MSDs. p: Probability value from chi-square analysis for the association between expectations and HCP status, which assessed the difference between HCP and Not-HCP. *p < 0.05.

Variable	HCP Status				Total		p
	HCP		Not-HCP				
	n	%	n	%	n	%	
Relaxing ambiance							0.076
Yes	42	75	23	92	65	80.25	
No	14	25	2	8	16	19.75	
Exceptional customer service							0.205
Yes	37	66.07	20	80	57	70.37	
No	19	33.93	5	20	24	29.63	
Healthy lounge							0.065
Yes	28	50	18	72	46	56.79	
No	28	50	7	28	35	43.21	
Premium materials							0.307
Yes	29	51.79	16	64	45	55.56	
No	27	48.21	9	36	36	44.44	
Synchronized scent, sound and visuals							0.001*
Yes	23	41.07	20	80	43	53.09	
No	33	58.93	5	20	38	46.91	
Hydrotherapy pools							0.618
Yes	28	50	11	44	39	48.15	
No	28	50	14	56	42	51.85	
Membership service and discounts							0.084
Yes	22	39.29	15	60	37	45.68	
No	34	60.71	10	40	44	54.32	
Sound-absorbing materials							0.561
Yes	23	41.07	12	48	35	43.21	
No	33	58.93	13	52	46	56.79	
Sauna							0.69
Yes	22	39.29	11	44	33	40.74	
No	34	60.71	14	56	48	59.26	
Healthy building certification							0.011*
Yes	15	26.79	14	56	29	35.8	
No	41	73.21	11	44	52	64.2	
Cryotherapy lounges							0.734
Yes	18	32.14	9	36	27	33.33	
No	38	67.86	16	64	54	66.67	
Meditation pods							0.061
Yes	15	26.79	12	48	27	33.33	
No	41	73.21	13	52	54	66.67	
Sound baths							0.087
Yes	14	25	11	44	25	30.86	
No	42	75	14	56	56	69.14	
Sustainable practices							0.001*
Yes	10	17.86	14	56	24	29.63	
No	46	82.14	11	44	57	70.37	
Crystal healing areas							0.023*
Yes	11	19.64	11	44	22	27.16	
No	45	80.36	14	56	59	72.84	
Light therapy							0.115
Yes	11	19.64	9	36	20	24.69	
No	45	80.36	16	64	61	75.31	
Other							0.064
Yes	7	12.5	0	0	7	8.64	
No	49	87.5	25	100	74	91.36	

Table 4 depicts the respondents' perceptions about the type of HCP they would be willing to seek MSD treatment from at a luxury resort hotel. For 3/8 HCP types (acupuncturist, chiropractor, pain management specialist), significant associations (p < 0.05) were found between HCP type and HCP status. For these three HCP types, a higher percentage of Not-HCPs reported "Yes" compared with HCPs, indicating that not-HCPs are more willing to seek MSD treatment from various HCP types in luxury resort hotels. More than 50% of Not-HCPs reported "Yes" to 6/8 HCP types: physical therapist (92%), massage therapist (88%), acupuncturist (72%), chiropractor (72%), pain management (72%), and psychologist (52%). In contrast, more than 50% of HCPs reported "Yes" to only 2/8 HCP types: physical therapist (82.14%) and massage therapist (69.64%).

**Table 4:** Respondents' perceptions about receiving services for MSDs at a luxury resort hotel from various healthcare professionals. Specific survey question: "What type of healthcare professional would you be willing to seek treatment from in a luxury resort hotel for an MSD? Select all that apply." Tabulation (n, %) of original survey data from respondents. Completion of the survey by the respondent was considered informed consent. HCP: Healthcare professional who manages musculoskeletal disorders (MSDs). Not-HCP: Member of the general public who does not manage MSDs. p: Probability value from chi-square analysis for the association between expectations and HCP status, which assessed the difference between HCP and Not-HCP. *p < 0.05.

Variable	HCP Status				Total		p
	HCP		Not-HCP				
	n	%	n	%	n	%	
Physical therapist							0.249
Yes	46	82.14	23	92	69	85.19	
No	10	17.86	2	8	12	14.81	
Massage therapist							0.077
Yes	39	69.64	22	88	61	75.31	
No	17	30.36	3	12	20	24.69	
Chiropractor							0.015*
Yes	24	42.86	18	72	42	51.85	
No	32	57.14	7	28	39	48.15	
Acupuncturist							0.010*
Yes	23	41.07	18	72	41	50.62	
No	33	58.93	7	28	40	49.38	
Psychologist							0.361
Yes	23	41.07	13	52	36	44.44	
No	33	58.93	12	48	45	55.56	
Pain management specialist							<0.001*
Yes	14	25	18	72	32	39.51	
No	42	75	7	28	49	60.49	
Family practice physician							0.571
Yes	12	21.43	4	16	16	19.75	
No	44	78.57	21	84	65	80.25	
Orthopaedic surgeon							0.552
Yes	7	12.5	2	8	9	11.11	
No	49	87.5	23	92	72	88.89	
Other							0.587
Yes	4	7.14	1	4	5	6.17	
No	52	92.86	24	96	76	93.83	

Table 5 depicts the respondents' likelihood of receiving MSD services at a luxury resort hotel and how much they would be willing to pay for an average daily rate for accommodation at a luxury resort hotel that provides MSD services. A significant association (p = 0.013) was found between the likelihood of receiving MSD services at a luxury resort hotel and HCP status. For example, a higher percentage of Not-HCPs reported they would likely receive MSD services at a luxury resort hotel compared with HCPs, as indicated by scores of 8, 9, or 10 on the 0-10 scale with anchors of 0 = not at all likely and 10 = extremely likely (Not-HCP 64%, HCP 21.43%). Moreover, a significant association (p = 0.028) was found between the average daily rate willing to pay and HCP status. For example, a higher percentage of HCPs selected the lowest range (< $900) for how much they would be willing to pay for the hotel ADR to receive MSD services (HCP 76.79%, Not-HCP 48%).

**Table 5:** Respondents' likelihood of receiving services for musculoskeletal disorders at a luxury resort hotel and their willingness to pay for these services. Specific survey questions: "How likely would you be to receive services for MSDs in a luxury resort hotel?" and "How much would you be willing to pay for an average daily rate for accommodation in a luxury resort hotel that provides services for MSDs?" Tabulation (n, %) of original survey data from respondents. Completion of the survey by the respondent was considered informed consent. HCP: Healthcare Professional who manages musculoskeletal disorders (MSDs). Not-HCP: Member of the general public who does not manage MSDs. p: Probability value from chi-square analysis for the association between expectations and HCP status, which assessed the difference between HCP and Not-HCP. *p < 0.05.

Variable	HCP Status				Total		p
	HCP		Not-HCP				
	n	%	n	%	n	%	
How likely would you be to receive services for MSDs in a luxury resort hotel?							0.013*
0 (Not at all likely)	8	14.29	1	4	9	11.11	
1	7	12.5	1	4	8	9.88	
2	7	12.5	0	0	7	8.64	
3	4	7.14	0	0	4	4.94	
4	0	0	1	4	1	1.23	
5	8	14.29	1	4	9	11.11	
6	4	7.14	1	4	5	6.17	
7	6	10.71	4	16	10	12.35	
8	6	10.71	10	40	16	19.75	
9	3	5.36	4	16	7	8.64	
10 (Extremely likely)	3	5.36	2	8	5	6.17	
How much would you be willing to pay for an average daily rate for accommodation in a luxury resort hotel that provides services for MSDs?							0.028*
< $900	43	76.79	12	48	55	67.9	
$1,000-$2,999	11	19.64	13	52	24	29.63	
$3,000-$4,999	1	1.79	0	0	1	1.23	
$5,000-$6,999	1	1.79	0	0	1	1.23	
$7,000-$8,999	0	0	0	0	0	0	
>$9,000	0	0	0	0	0	0	

The respondents indicated that they would be willing to pay a 32.3 ± 29.2% premium above the average daily rate for accommodation to receive MSD services at a luxury resort hotel, with no significant difference (p = 0.396) observed between HCPs and Not-HCPs. Additionally, the respondents indicated that they would be willing to stay in a luxury resort hotel for 5.8 ± 3.9 days to receive MSD services, with Not-HCPs willing to stay significantly longer (p = 0.048) than HCPs (Not-HCP 7.0 ± 3.9 days, HCP 5.2 ± 3.9 days).

Two survey items were specifically intended for HCPs. 43% of HCPs indicated that they are likely to deliver MSD services in a luxury resort hotel vs 36% unlikely, as indicated by scores of 8, 9, or 10 categorized as likely and scores of 0-2 categorized as unlikely on the 0-10 scale (0 = not at all likely, 10 = extremely likely). The HCPs also indicated that they would be willing to accept $254.40 ± $151.50 (median: $249.50) per hour to deliver MSD services at a luxury resort hotel.

## Discussion

Key findings

The present study was the first known attempt to assess the preferences, attitudes, and expectations of HCPs and Not-HCPs about receiving MSD services in luxury resort hotels compared with clinicians' offices. While the study's limitations, as noted herein, preclude widespread generalizability, the findings of this study help fill knowledge gaps and inform future research and implementation efforts about healthcare service delivery in non-traditional settings for MSDs. As previously detailed in this article, the ongoing burden of MSDs and challenges to manage them are large [1-3,13,14]. Thus, innovative delivery approaches should be explored [19], such as those examined in the present study.

Overall, 84% of respondents of the present study reported that they sought treatment from a HCP for MSDs over their lifetime, which appears to be high. While no data are available to make direct comparisons with this utilization rate, previous work indicates that healthcare utilization for MSDs is variable and can be high [44-46]. Regardless of variability, the observed 84% utilization rate suggests that respondents had pragmatic experiences with MSDs that were relevant to the present study.

The respondents of the present study indicated that they were generally willing to receive care for MSDs in luxury resort hotels compared with clinicians' offices. Notably, a high percentage of participants indicated that receiving care for MSDs at a luxury resort hotel would be more comfortable and enjoyable, less intimidating, and more likely to result in adherence to therapeutic exercise and improvement in overall health and wellness. Additionally, the respondents identified potential barriers and facilitators to seeking MSD services in luxury resort hotels, such as affordability, proximity, competence of HCPs, and educational aspects that align with patient-centered care delivery models [47]. This study also found that Not-HCPs had more favorable views than HCPs about receiving MSD services in luxury resort hotels across nearly every survey item that assessed this concept. In addition, HCPs expressed ambivalence about delivering MSD services at luxury resort hotels, which appears to be consistent with their views about receiving these services.

Limitations

The limitations of the present study were predominantly related to its exploratory design. The study utilized convenience sampling and snowball sampling strategies that are not reproducible by other investigators and resulted in enrollment of a small sample of participants who were not randomly selected or controlled. Moreover, the sample may not have been representative of HCPs who manage MSDs and members of the general public who do not manage MSDs (Not-HCPs), and the specific professional backgrounds and sub-groups of the HCP respondents are unknown. Also, the study sample may have had an over-representation of physical therapists since the sample was derived from the investigator's mailing list. Furthermore, the questionnaire used in the study was an exploratory, non-validated instrument; thus, its psychometric properties and ability to accurately examine preferences, attitudes, and expectations are unknown. Finally, the study has inherent limitations that are associated with cross-sectional survey research designs. Namely, cross-sectional surveys rely on self-reported perceptions, not validated outcomes that can be used as evidence to assess the effectiveness of MSD interventions. Considering these limitations, the results of this study could be prone to a high risk of bias and low generalizability (i.e., poor external validity) [39,48].

Interpretations

Considering the limitations of the present study, its results should be interpreted with caution. First, the factors affecting why respondents, particularly Not-HCPs, expressed willingness to receive MSD care in luxury resort hotels are unclear. It is plausible that respondents perceived luxury resort hotels as viable options to receive care. Alternatively, these perceptions may represent an overall dissatisfaction with traditional delivery settings (i.e., clinicians' offices), providers, and therapeutic outcomes. Furthermore, these findings may indicate miscommunication and misalignment of values and expectations between clinicians (supply) and patients (demand). This explanation is supported by a systematic review that highlighted areas of patients' dissatisfaction with healthcare providers and other aspects of service delivery for MSDs [16]. This previous review also noted the patients' desires for patient-centered care, open communication with providers, strong education about their condition, and holistic care [16], which are aligned with the findings of the present study.

In the present study, 68% (17/25) of Not-HCPs answered "agree" or "strongly agree" to the survey item, "I believe that I am more likely to adhere to exercise recommendations at a luxury resort hotel than a clinician's office." This finding is consistent with previous work indicating that many patients with low back pain do not adhere to therapeutic exercise programs delivered in clinicians' offices [14]. While the present study did not assess actual treatment adherence or behavioral outcomes, it is plausible that patients may be seeking settings that they believe are conducive to therapeutic exercise, for which adherence is associated with various intrapersonal, interpersonal, and administrative factors [49].

The respondents also indicated that affordability is an important consideration for seeking care for MSDs in luxury resort hotels. This finding is consistent with previous work reporting that patients with low back pain who paid more than $40 per visit out of pocket were nearly 3.5 times less likely to adhere to treatment compared with those who were not required to pay anything out of pocket [50]. While examining the specific cost-effectiveness threshold for treatment is beyond the scope of the present study, it is possible that the influence of payment mechanism (self-pay versus third-party payor) is an important consideration regarding MSD care. Hence, health economic evaluations involving various stakeholders should be conducted for delivering MSD services in alternative settings, such as luxury resort hotels.

Future research

Future research is needed to address the limitations described above in order to build upon previous research [19], and make meaningful contributions to advance this new field. A definitive survey instrument should be developed utilizing recommended approaches [39], along with formal consensus processes such as the modified Delphi process that has been used for other aspects of MSD management [51]. The psychometric properties (e.g., reliability and validity) of the survey instrument should be examined. Various types of validity, such as content, criterion, and construct validity [52], should be assessed before this instrument can be used to inform clinical decision-making for the management of MSDs in non-traditional settings. Subsequently, the study should be replicated in representative samples of participants across various settings to enhance generalizability.

In addition to survey research, future prospective and longitudinal studies are needed to examine the characteristics of patients who may be best suited for receiving MSD services in luxury resort hotels, as well as the characteristics of HCPs who may be most appropriate to deliver these services. Also, adequately powered controlled trials and implementation research studies using validated clinical outcomes are needed to compare the effectiveness of receiving and delivering MSD services in luxury resort hotels versus clinicians' offices. Finally, other non-traditional approaches for MSD healthcare service delivery should be explored.

Pragmatic applications

The main pragmatic application of the present study is to inform future research, as discussed herein. Speculating on the direct impact of the present study on specific pragmatic applications for healthcare delivery for MSDs is beyond the scope of this study since its results and interpretations rely on qualitative perceptions, not standard clinical outcomes. Nonetheless, if positive findings are observed in future investigations, delivery of services for MSDs in non-traditional settings, such as luxury resort hotels, may have the potential to improve the quality of life of some patients suffering from MSDs, as well as create a new healthcare value proposition for various stakeholders [19].

## Conclusions

In summary, the present study, which surveyed a convenience sample of 81 participants, was the first known attempt to assess the preferences, attitudes, and expectations of HCPs and Not-HCPs about receiving MSD services in luxury resort hotels compared with clinician offices. The findings suggest that Not-HCPs had more favorable views than HCPs about receiving MSD care in luxury resort hotels, and HCPs were hesitant about delivering MSD services in this non-traditional setting. However, due to a small sample size and the exploratory, non-validated nature of the survey instrument, the results of this study may be prone to high risk of bias and low generalizability. While future research is needed to inform clinical practice, this study provides insights about potential misalignments between perceptions of patients and HCPs regarding non-traditional delivery settings for the management of MSDs.

## Appendices

**Table 6:** Supplemental Table: Checklist for Reporting of Survey Studies.

Section/topic	Item	Item description	Reported in section
Title and abstract			
Title and abstract	1a	State the word “survey” along with a commonly used term in title or abstract to introduce the study’s design.	Title
	1b	Provide an informative summary in the abstract, covering background, objectives, methods, findings/results, interpretation/discussion, and conclusions.	Abstract
Introduction			
Background	2	Provide a background about the rationale of study, what has been previously done, and why this survey is needed.	Introduction, paragraphs 1-6
Purpose/aim	3	Identify specific purposes, aims, goals, or objectives of the study.	Introduction, paragraph 7
Methods			
Study design	4	Specify the study design in the methods section with a commonly used term (e.g., cross-sectional or longitudinal).	Materials & Methods, Study Design, paragraph 1
	5a	Describe the questionnaire (e.g., number of sections, number of questions, number and names of instruments used).	Materials & Methods, Data Collection Methods, paragraphs 1-3
Data collection methods	5b	Describe all questionnaire instruments that were used in the survey to measure particular concepts. Report target population, reported validity and reliability information, scoring/classification procedure, and reference links (if any).	Materials & Methods, Data Collection Methods, paragraphs 1-3
	5c	Provide information on pretesting of the questionnaire, if performed (in the article or in an online supplement). Report the method of pretesting, number of times questionnaire was pre-tested, number and demographics of participants used for pretesting, and the level of similarity of demographics between pre-testing participants and sample population.	Materials & Methods, Data Collection Methods, paragraph 3
	5d	Questionnaire if possible, should be fully provided (in the article, or as appendices or as an online supplement).	Appendix
Sample characteristics	6a	Describe the study population (i.e., background, locations, eligibility criteria for participant inclusion in survey, exclusion criteria).	Materials & Methods, Sample Characteristics, paragraphs 1-3
	6b	Describe the sampling techniques used (e.g., single stage or multistage sampling, simple random sampling, stratified sampling, cluster sampling, convenience sampling). Specify the locations of sample participants whenever clustered sampling was applied.	Materials & Methods, Sample Characteristics, paragraphs 1-3
	6c	Provide information on sample size, along with details of sample size calculation.	Materials & Methods, Sample Characteristics, paragraphs 1-3
	6d	Describe how representative the sample is of the study population (or target population if possible), particularly for population-based surveys.	Materials & Methods, Sample Characteristics, paragraphs 1-3
Survey administration	7a	Provide information on modes of questionnaire administration, including the type and number of contacts, the location where the survey was conducted (e.g., outpatient room or by use of online tools, such as SurveyMonkey).	Materials & Methods, Survey Administration, paragraphs 1-2
	7b	Provide information of survey’s time frame, such as periods of recruitment, exposure, and follow-up days.	Materials & Methods, Survey Administration, paragraph 1
	7c	Provide information on the entry process: –>For non-web-based surveys, provide approaches to minimize human error in data entry. –>For web-based surveys, provide approaches to prevent “multiple participation” of participants.	Materials & Methods, Survey Administration, paragraph 2
Study preparation	8	Describe any preparation process before conducting the survey (e.g., interviewers’ training process, advertising the survey).	Materials & Methods, Data Collection Methods, paragraphs 1-3
Ethical considerations	9a	Provide information on ethical approval for the survey if obtained, including informed consent, institutional review board [IRB] approval, Helsinki declaration, and good clinical practice [GCP] declaration (as appropriate).	Materials & Methods, Ethical Considerations, paragraph 1
	9b	Provide information about survey anonymity and confidentiality and describe what mechanisms were used to protect unauthorized access.	Materials & Methods, Ethical Considerations, paragraph 1
Statistical analysis	10a	Describe statistical methods and analytical approach. Report the statistical software that was used for data analysis.	Materials & Methods, Analysis, paragraph 1
	10b	Report any modification of variables used in the analysis, along with reference (if available).	Materials & Methods, Analysis, paragraph 1
	10c	Report details about how missing data was handled. Include rate of missing items, missing data mechanism (i.e., missing completely at random [MCAR], missing at random [MAR] or missing not at random [MNAR]) and methods used to deal with missing data (e.g., multiple imputation).	Materials & Methods, Analysis, paragraph 1, Results, Respondent Characteristics, paragraphs 1-3
	10d	State how non-response error was addressed.	Results, Respondent Characteristics, paragraphs 1-3
	10e	For longitudinal surveys, state how loss to follow-up was addressed.	Not applicable
	10f	Indicate whether any methods such as weighting of items or propensity scores have been used to adjust for non-representativeness of the sample.	Not applicable
	10g	Describe any sensitivity analysis conducted.	Not applicable
Results			
Respondent characteristics	11a	Report numbers of individuals at each stage of the study. Consider using a flow diagram, if possible.	Results, Respondent Characteristics, paragraphs 1-2, Figure 1
	11b	Provide reasons for non-participation at each stage, if possible.	Not applicable.
	11c	Report response rate, present the definition of response rate or the formula used to calculate response rate.	Results, Respondent Characteristics, paragraph 1
	11d	Provide information to define how unique visitors are determined. Report number of unique visitors along with relevant proportions (e.g., view proportion, participation proportion, completion proportion).	Results, Respondent Characteristics, paragraphs 1-3
Descriptive results	12	Provide characteristics of study participants, as well as information on potential confounders and assessed outcomes.	Results, Respondent Characteristics, paragraphs 1-3, Descriptive Results, paragraphs 1-2
Main findings	13a	Give unadjusted estimates and, if applicable, confounder-adjusted estimates along with 95% confidence intervals and p-values.	Results, Main Results, Tables 1-5
	13b	For multivariable analysis, provide information on the model building process, model fit statistics, and model assumptions (as appropriate).	Results, Main Results, Tables 1-5
	13c	Provide details about any sensitivity analysis performed. If there are considerable amount of missing data, report sensitivity analyses comparing the results of complete cases with that of the imputed dataset (if possible).	Not applicable
Discussion			
Limitations	14	Discuss the limitations of the study, considering sources of potential biases and imprecisions, such as non-representativeness of sample, study design, important uncontrolled confounders.	Discussion, Limitations, paragraph 1
Interpretations	15	Give a cautious overall interpretation of results, based on potential biases and imprecisions and suggest areas for future research.	Discussion, Key Findings, paragraphs 1-3, Interpretation, paragraphs 1-3
Generalizability	16	Discuss the external validity of the results.	Discussion, Key Findings, paragraph 1, Limitations, paragraph 1, Future Research, paragraph 1
Other sections			
Role of funding source	17	State whether any funding organization has had any roles in the survey’s design, implementation, and analysis.	Submission site field, Funding details
Conflict of interest	18	Declare any potential conflict of interest.	Submission site field, Disclosure Statement / Conflict of Interest
Acknowledgements	19	Provide names of organizations/persons that are acknowledged along with their contribution to the research.	Submission site field, Acknowledgements

Appendix: Survey Instrument

Title: Where Do I Prefer to Receive Care for Musculoskeletal Disorders? A Survey Questionnaire.

Instructions:

Please complete the following 13 items of this survey questionnaire. Item numbers 1 through 5, and 8 through 13 allow one response. Items 6 and 7 allow multiple responses. Please select the response(s) that most accurately reflect your thoughts, feelings, and preferences right now. Please complete the survey questionnaire only one time.

Each item focuses on musculoskeletal disorders (MSDs), clinicians' offices, and luxury resort hotels.

MSDs are: "Conditions that affect the muscles, bones, joints, tendons, ligaments, and nerves." Back pain, neck pain, shoulder pain, hip pain, and knee pain are examples of MSDs.

A clinician's office is: The traditional location where a "health professional works with patients to diagnose or treat illnesses."

A luxury resort hotel is: "A high-end, upscale hotel that is situated in a resort location, offering not only luxurious accommodations but also a wide range of recreational activities, exceptional service, fine dining, and often unique amenities, all designed to provide a premium vacation experience for guests seeking a lavish getaway."

Consent language (abridged): Your participation will be considered your consent, no personally identifiable information will be collected, and your responses will be kept confidential. Your participation in this study is voluntary and will take approximately 10 to 15 minutes. If you choose not to participate or withdraw from the study at any time, there will be no penalty. You have the right not to answer any questions, and you can stop the study at any time. You must be 18 or older to participate.

The results of the study may be published in an academic journal, but no personally identifiable information will be known. Please do not put your name or any other personally identifiable information on the survey questionnaire. You, the participant, will be anonymous to our research. Additionally, results will only be shared in the aggregate form; de-identified data collected from the current study will not be shared with other investigators for future research purposes. There are no foreseeable risks or discomforts in your participation.

Completion of the survey questionnaire will be considered your consent to participate. If you have any questions concerning the survey questionnaire, please contact <redacted>.

1. What country do you reside in?

<Drop-down menu>

2. Are you a healthcare professional (HCP) who manages MSDs?

Yes

No

3. Have you ever received services from an HCP for an MSD?

Yes

No

4. Select the response that most accurately represents your expectations about receiving services for an MSD. When answering these items, assume that each type of facility listed below is equal in terms of having appropriately trained staff, licensed and experienced healthcare professionals, rehabilitation equipment, and other resources needed to deliver services safely, along with similar costs.

4.1. I would rather go to a luxury resort hotel for treatment of an MSD than a clinician's office.

Strongly disagree

Disagree

Neither agree nor disagree

Agree

Strongly agree

4.2. I believe that a luxury resort hotel is more convenient for me than a clinician's office.

Strongly disagree

Disagree

Neither agree nor disagree

Agree

Strongly agree

4.3. I believe that a luxury resort hotel will be more comfortable for me than a clinician's office.

Strongly disagree

Disagree

Neither agree nor disagree

Agree

Strongly agree

4.4. I believe that a luxury resort hotel will be less intimidating for me than a clinician's office.

Strongly disagree

Disagree

Neither agree nor disagree

Agree

Strongly agree

4.5. I believe that a luxury resort hotel is more likely to help my disability and pain than a clinician's office.

Strongly disagree

Disagree

Neither agree nor disagree

Agree

Strongly agree

4.6. I believe that a luxury resort hotel is more likely to improve my overall health and wellness than a clinician's office.

Strongly disagree

Disagree

Neither agree nor disagree

Agree

Strongly agree

4.7. I believe that I am more likely to adhere to therapeutic exercise recommendations at a luxury resort hotel than in a clinician's office.

Strongly disagree

Disagree

Neither agree nor disagree

Agree

Strongly agree

4.8. I would more likely enjoy the experience of receiving MSD services at a luxury resort hotel than a clinician's office.

Strongly disagree

Disagree

Neither agree nor disagree

Agree

Strongly agree

5. How important are each of these items for you when making decisions about receiving services for MSDs at a luxury resort hotel?

5.1. Proximity (i.e., travel time) of the hotel to my residence.

Not important at all

Slightly important

Of average importance

Very important

5.2. Receiving some type of incentives for these services.

Not important at all

Slightly important

Of average importance

Very important

5.3. Understanding if my insurance covers these services or if they are paid for out of my pocket.

Not important at all

Slightly important

Of average importance

Very important

5.4. Being able to afford these services.

Not important at all

Slightly important

Of average importance

Very important

5.5. Understanding the recovery time from an MSD to estimate my length of stay at the luxury resort hotel.

Not important at all

Slightly important

Of average importance

Very important

5.6. Determining how to find a luxury resort hotel that provides musculoskeletal services.

Not important at all

Slightly important

Of average importance

Very important

5.7. Assessing the caliber of the healthcare professionals providing musculoskeletal services.

Not important at all

Slightly important

Of average importance

Very important

5.8. Understanding if my musculoskeletal condition can be treated at a luxury resort hotel.

Not important at all

Slightly important

Of average importance

Very important

6. What features and services do you expect to see at a luxury resort hotel that you will not find at a clinician's office when receiving musculoskeletal services? Select all that apply.

6.1. Premium materials

Yes

No

6.2. Sound-absorbing materials

Yes

No

6.3. Relaxing ambiance

Yes

No

6.4. Synchronized scent, sound, and visuals

Yes

No

6.5. Hydrotherapy pools

Yes

No

6.6. Cryotherapy lounges

Yes

No

6.7. Sauna

Yes

No

6.8. Sound baths

Yes

No

6.9. Light therapy

Yes

No

6.10. Meditation pods

Yes

No

6.11. Crystal healing areas

Yes

No

6.12. Sustainable practices

Yes

No

6.13. Exceptional customer service

Yes

No

6.14. Membership service and discounts

Yes

No

6.15. Healthy lounge

Yes

No

6.16. Healthy building certification

Yes

No

6.17. Other

Yes

No

Fill in (text):

7. What type of healthcare professional would you be willing to seek treatment from in a luxury resort hotel for an MSD? Select all that apply.

7.1. Physical therapist

Yes

No

7.2. Chiropractor

Yes

No

7.3. Family practice physician

Yes

No

7.4. Orthopaedic surgeon

Yes

No

7.5. Pain management

Yes

No

7.6. Psychologist

Yes

No

7.7. Acupuncturist

Yes

No

7.8. Massage therapist

Yes

No

7.9. Other

Yes

No

Fill in (text):

8. How likely would you be to receive services for MSDs in a luxury resort hotel?

0 (Not at all likely), 1, 2, 3, 4, 5, 6, 7, 8, 9, 10 (Extremely likely)

9. How much would you be willing to pay for an average daily rate for accommodation in a luxury resort hotel that provides services for MSDs?

<$900

$1,000-$2,999

$3,000-$4,999

$5,000 -$6,999

$7,000 -$8,999

>$9,000

10. How much of a premium would you be willing to pay for receiving services for musculoskeletal disorders in a luxury resort hotel, in addition to the daily accommodation rate?

Line slider with percentages ranging from 0 to 200%

11. How long would you be willing to stay in a luxury resort hotel to receive professional healthcare services?

Line slider with days ranging from 0 to 21 days

Display items 12 and 13 if Yes is selected for Item 2: Are you a healthcare professional who manages MSDs?

12. How likely would you be to deliver services for MSDs in a luxury resort hotel?

0 (Not at all likely), 1, 2, 3, 4, 5, 6, 7, 8, 9, 10 (Extremely likely)

13. How much would you be willing to accept for an hourly rate to deliver services to MSDs in a luxury resort hotel?

Line slider with US dollars ranging from $0 to $1000
